# Long-Term Dietary and Physical Activity Interventions in the School Setting and Their Effects on BMI in Children Aged 6–12 Years: Meta-Analysis of Randomized Controlled Clinical Trials

**DOI:** 10.3390/healthcare9040396

**Published:** 2021-04-01

**Authors:** Purificación Cerrato-Carretero, Raúl Roncero-Martín, Juan D. Pedrera-Zamorano, Fidel López-Espuela, Luis M. Puerto-Parejo, Antonio Sánchez-Fernández, María Luz Canal-Macías, Jose M. Moran, Jesus M. Lavado-García

**Affiliations:** 1 Metabolic Bone Diseases Research Group, Nursing Department, Nursing and Occupational Therapy College, University of Extremadura, 10003 Caceres, Spain; puricc@yahoo.es (P.C.-C.); rronmar@unex.es (R.R.-M.); jpedrera@unex.es (J.D.P.-Z.); fidellopez@unex.es (F.L.-E.); lmpuerto@unex.es (L.M.P.-P.); luzcanal@unex.es (M.L.C.-M.); jmlavado@unex.es (J.M.L.-G.); 2 Gynecology Department, Complejo Hospitalario Universitario de Cáceres, 10003 Caceres, Spain; gineantonio@gmail.com

**Keywords:** meta-analysis, body mass index, school children, physical activity, health education, dietary interventions

## Abstract

Preventive actions and potential obesity interventions for children are mainly researched throughout the school period, either as part of the school curricula or after regular school hours, via interventions mostly lasting less than 12 months. We aimed to perform a meta-analysis on randomized controlled clinical trials to evaluate the evidence of the efficacy of long-term school-based interventions in the management of childhood obesity in terms of BMI from a dietary and physical activity-based approach. Eleven randomized controlled clinical trials were examined using the random effects model, and the results showed that there were no significant effects associated with physical activity + nutrition intervention in school children aged 6–12 years, with a pooled standardized mean difference (SMD) (95% CI) of −0.00 (−0.05, 0.04). No effects were observed after subgroup analysis based on the intervention length. The findings from our study indicate that long-term school-based interventions on physical activity and dietary habits received by children aged 6–12 years seem to have no effect on BMI. However, the promotion of such interventions should not be discouraged, as they promote additional positive health outcomes for other domains of children’s health.

## 1. Introduction

Health risks associated with childhood obesity are a critical challenge for public health in the 21st century [1]. Obesity in children is a major global public health risk with overweight and obesity rates representing a serious public health problem, with prevalence rates among U.S. schoolchildren reaching 31.8% for overweight standards and 16.9% for obesity in 2011–2012 [2] and increasing to 18.5% in 2017–2018 [3]. Similar figures have been reported in Canada [4] and European countries [5].

Interventions designed to target the overweight and obesity problem in children have been implemented from the point of view of school, home, primary care, childcare, community and consumer health informatics approaches. Preventive actions and potential obesity interventions for children are mainly researched throughout the school period, either as part of the school curricula or after regular school hours [6], via interventions mostly lasting less than 12 months. The school environment gives access to a number of subjects who interact continuously and actively with this environment in the early decades of their lives, enabling them to access physical education programs, classroom health education and school health services [1]. Nevertheless, studies conducted to evaluate interventions in the school setting [7,8,9] have shown conflicting results on the potential efficacy of these interventions to improve body mass index, some of which demonstrate weak efficacy in trials that assessed a combination of nutrition plus physical activity (standardized mean difference −0.29, 95% confidence interval (CI): −0.45 to −0.14) [10] and mostly show small [11,12] or no effects [13,14,15].

The literature is abundant in analyzing school interventions based on physical activity, and also in analyzing the influence of dietary interventions in the same setting upon BMI. Therefore, this is the starting point of our work, since the assessment of the joint effect of both types of interventions has been less studied. As an academic year has been proposed as a reference point [11,16] from which positive effects are observed on the BMI of children subjected to this type of intervention we aimed to identify and perform a meta-analysis on randomized controlled clinical trials to evaluate the evidence of the efficacy of long-term school-based interventions in the management of childhood obesity in terms of BMI and from a dietary and physical activity-based approach.

## 2. Materials and Methods

This meta-analysis was conducted in accordance with the Preferred Reporting Items for Systematic Reviews and Meta-Analyses (PRISMA) guidelines [17] (Supplementary Table S1) as well as the recommendations of the Cochrane Collaboration Handbook [18].

### 2.1. Search Strategy

An independent literature review was completed by two researchers in the following databases: MEDLINE (via PubMed), Scopus, and Web of Science. Articles that considered the impact of school-based physical activity plus diet or dietary education interventions on BMI in children aged 6–12 years and that were supported by randomized controlled clinical trials were included. In accordance with the PRISMA recommendations, the search strategy used for PubMed/MEDLINE is shown in Table 1. The bibliographic references listed in the studies of interest were manually searched to identify additional eligible trials. In accordance with this approach, an additional study was included in the analysis [19].

The complete strategies for WOS and Scopus are summarized in Supplementary Table S2.

### 2.2. Study Selection

Studies fulfilling the following eligibility criteria were included: (i) children aged 6–12 years (grades 3–8); (ii) physical activity interventions in combination with dietary interventions or dietary education compared to a no-intervention control group receiving no treatment or routine care or any other active interventions; (iii) interventions of at least one year or a full academic year in duration; (iv) randomized controlled trial (RCT); (v) studies reporting BMI before and after intervention or BMI after intervention substantiating no statistically significant difference at baseline; (vi) studies written in English; and (vii) studies published within the last 10 years (until December 2019). The exclusion criteria were as follows: (i) BMI was not objectively determined but instead reported by the participants themselves; (ii) RCTs designed to treat childhood obesity or eating disorders or clinical cohorts; (iii) those that included drug treatments or surgical interventions; and (iv) those that included dietary supplements.

After excluding duplicate studies across the different databases, the titles and abstracts of the identified articles were evaluated by two investigators (P.C.-C. and J.M.M.) to identify eligible trials. Should the content of the abstract indicate incomplete fulfillment of the inclusion or exclusion criteria, content analysis was performed by reading the full text. Subsequently, the reasons for exclusion and inclusion of the different studies were discussed. Where disagreement arose, a third investigator (R.R.-M.) made the final decision.

### 2.3. Data Synthesis

The primary outcome measurement was the mean change in BMI, calculated as the postintervention mean BMI minus the preintervention mean BMI. BMI has been used as a primary outcome measure in numerous studies given that it is a reliable determinant of general adiposity, superior to the BMI Z-score [15,20,21,22], and is routinely used and reported in studies investigating the effect of interventions for childhood obesity [23,24,25,26].

### 2.4. Assessment of Risk of Bias in Included Studies

The risk of bias of the included RCTs was assessed using the “Risk of Bias” tool [27]. Each trial was assessed by a minimum of two investigators as having a “high”, “low”, or “unclear” risk of bias for each guideline-considered element. Disagreements were addressed by discussion and consensus. Performance and detection bias were incorporated under the “blinding” item of the “Risk of Bias” tool. Generally, trials that showed sufficient information on the blinding of outcome assessors were considered to have a low risk of bias, and those that reported that outcome assessors were not blinded were considered to have a high risk of bias.

Trials were considered to have a low risk of attrition bias if an appropriate narrative of participant flow throughout the study was reported, and the rate of missing outcome data was sufficiently proportionate between groups and well substantiated. An attrition rate above 30% was considered high risk.

We assessed selective outcome reporting by retrieving the registries or prepublished protocols of the different trials. Discrepancies in the primary outcomes were considered high risk. Trials reporting an outcome not prespecified in the corresponding registry or protocol were considered high risk. When the registry or protocol of the trial could not be retrieved, it was considered to have an uncertain risk of bias.

Overall, when a study did not include sufficient information to allow a decision to be made regarding the domains assessed, an unclear evaluation was assigned.

### 2.5. Statistical Analysis

The combined standardized mean difference (SMD) between the educational interventions and the control groups was compared using random effects, with the results of the random effects model being those presented. Data were included only for outcomes reported immediately after the intervention. Postintervention follow-up data were not analyzed if they had been reported in the trial. Given that more than 10 studies were finally included in the meta-analysis, publication bias was evaluated by funnel plot asymmetry and tested using Egger’s test [28]. The I^2^ statistic was used to evaluate heterogeneity: I^2^ ranging from 0% to 40% indicated that heterogeneity was not relevant; I^2^ ranging from 30% to 60% indicated moderate heterogeneity; I^2^ ranging from 50% to 90% indicated substantial heterogeneity; and I^2^ ranging from 75% to 100% indicated considerable heterogeneity [29].

## 3. Results

### 3.1. Study Descriptions

**Figure 1:**
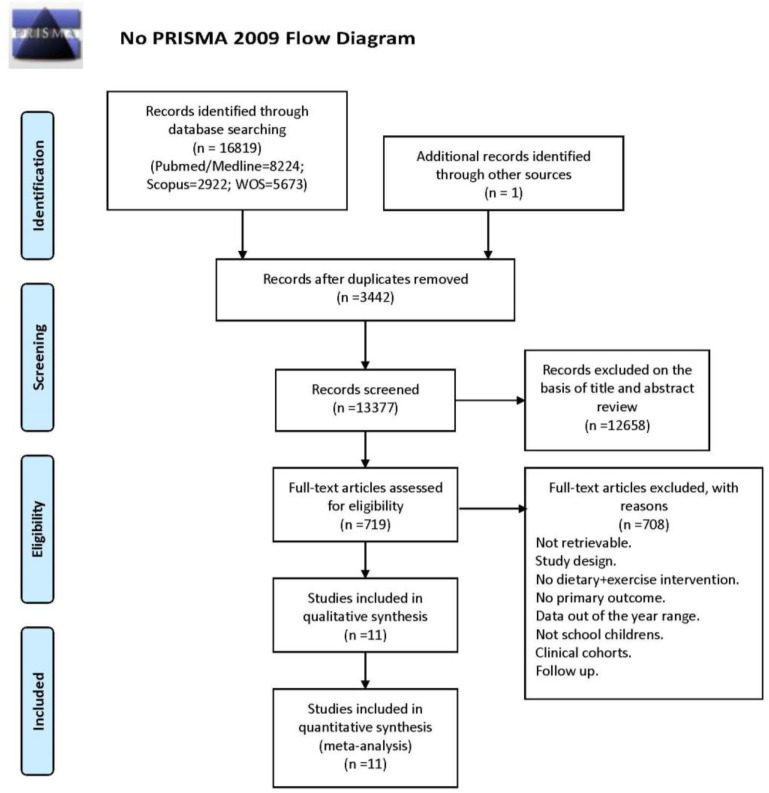
PRISMA flow diagram.

A search of trials published up to 10 years prior to December 2019 yielded a total of records. After reviewing the titles and abstracts, 719 of these records were subjected to full-text review read in their entirety, and 11 RCTs were ultimate included in the meta-analysis. Figure 1 shows the PRISMA flowchart for study selection.

Details of each of the studies included in the systematic review and meta-analysis are listed in Table 2, including data on the theory supporting the intervention, background, age, and country. Accordingly, our current meta-analysis comprises data retrieved from 11 published studies [19,30,31,32,33,34,35,36,37,38,39]. Two of the studies were treated as two different groups for the analyses performed in the present meta-analysis since the trials analyzed samples separately according to gender [35] and depending on the nature of the intervention [19]. The control group (which was unique) was, therefore, divided into both cases for the analysis.

### 3.2. Characteristics of the Randomized Controlled Trials Included

The 11 cluster RCTs included herein were undertaken in 8 different countries, most in Europe (*n* = 6) [30,31,32,33,36,38], South America [19,35], Asia [37,39] and Oceania [34]. Altogether, the 11 cluster RCTs involved individuals. RCTs covered in the present review were conducted between 2013 and 2019, with a reported median duration of 1.56 years (range 1–3.5).

### 3.3. BMI and Physical Activity + Nutrition Interventions

**Figure 2:**
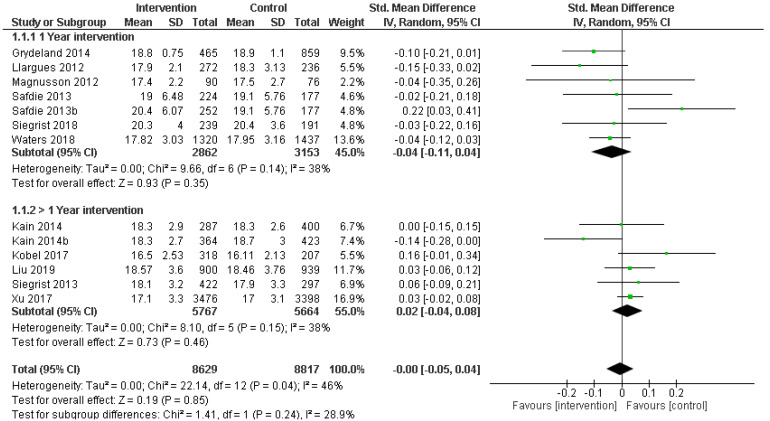
Forest plot of comparison: randomized controlled trial PA + dietary intervention versus control. Standardized mean difference in BMI from baseline to post-intervention.

Mean and SD values of BMI after physical activity + nutrition interventions (≥12 months) in school children aged 6–12 and control groups were pooled to obtain a total estimate of the overall effect. Moderate heterogeneity was found across studies (I^2^ = 46%; *p* = 0.04); thus, the random effects model was selected to report the pooled effect size. The results based on the random effects model showed that there were no significant effects associated with physical activity + nutrition intervention in school children. The pooled SMD and (95% CI) were −0.00 (−0.05, 0.04). The overall effect size for SMD calculated as Z was 0.19 (*p* = 0.85) (Figure 2). For a more detailed analysis, the trials were grouped according to their duration in 1 year (one school year) or with a duration longer than one school year. We found that children who received interventions with a duration of one academic year (1 year) had no statistically significant differences from controls after pooling these trials (SMD: −0.04; 95% CI: −0.11, 0.04; *p* = 0.35; Figure 2).

No significant heterogeneity was observed among the studies (I^2^ = 38%; *p* = 0.14). Subgroup analysis for those trials that explored interventions longer than 1 academic year also failed to observe statistically significant differences between children involved in the interventions and control children from baseline to the end of the trials for BMI (SMD: 0.02; 95% CI: −0.04, 0.08; *p* = 0.46; Figure 2). No significant heterogeneity was observed among these studies (I^2^ = 38%; *p* = 0.15)

### 3.4. Risk of Bias

**Figure 3:**
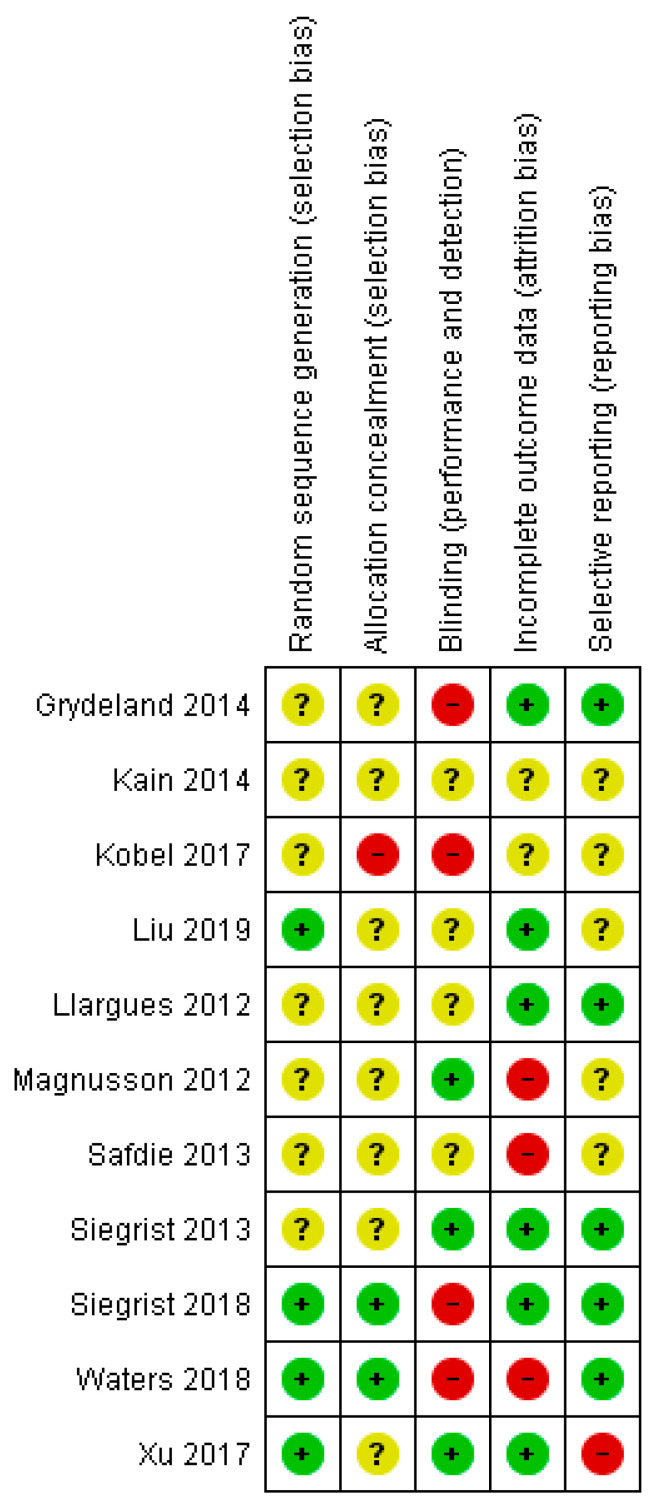
Risk of bias summary: review authors’ judgements about each risk of bias item for each included study.

Potential sources of bias were reviewed in five settings (Figure 3). Most studies (7 of 11) were classified as having an unclear risk of bias for random sequence generation (selection bias), while none were classified as having a high risk. While two studies were rated as having a low risk of allocation concealment (selection bias), the majority of studies were rated as having an unclear risk of underreporting.

**Figure 4:**
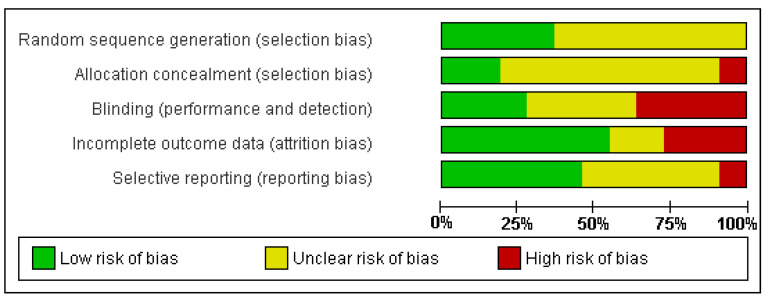
Risk of bias graph: review authors’ judgements about each risk of bias item presented as percentages across all included studies.

Authentic blinding of participants and personnel (implementation bias) may not have been fully practicable due to the nature of the designs of the studies, with 36.36% of the trials judged as having a high risk of bias in blinding of participants and/or personnel because such blinding was usually not possible for interventions of this nature. Most of the studies were assessed as having a low risk of attrition bias (*n*  =  6.54.5%). Only one study was assessed as having the possibility of selective reporting (*n*  =  1.9%) (Figure 4).

### 3.5. Publication Bias

**Figure 5:**
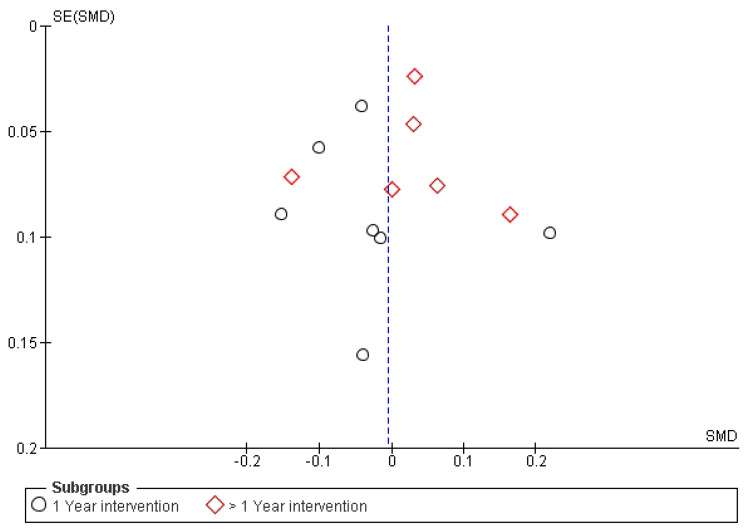
Funnel plot of standard error by SMD.

The funnel plot was rather symmetrical, suggesting that there was little risk of publication bias (Figure 5). Egger’s regression test (intercept = −0.2701; 95% CI −2.0219 to 1.4817; *p* = 0.7407), and Begg’s test (Kendall’s Tau = 0.07692; *p* = 0.7143) also suggested no publication bias among these studies.

## 4. Discussion

This review is one of the first to use meta-analyses to systematically review recent studies and to analyze long-term physical education plus dietary interventions in schoolchildren and their effect on BMI. One academic year has been proposed as the length of the intervention that is likely to have a positive effect on the evolution of BMI [16]. To the current body of knowledge, no significant associations were found between physical activity interventions in conjunction with long-term dietary interventions and BMI in the school setting. Our result is consistent with that of others who have also reviewed similar interventions at the school level [40,41] and inconsistent with other reviews, most likely because of differences in the target age and in the length of the proposed interventions [10]. Thus, schoolchildren who participate in combined physical activity and dietary interventions benefit from these when the interventions are of longer duration, while studies with a shorter duration appear to have no statistically significant results [42]. These results, apparently contrary to those obtained in our study, were reported after meta-analyzing studies that used overweight or obese schoolchildren as samples and, therefore, with interventions specifically focused on improving the prevalence of obesity and overweight in these populations. In these population groups, it is known that interventions may reflect a tendency to respond favorably in some children, particularly those who are obese or overweight, which could lead to a disproportionate improvement in BMI behavior in these groups [11]. The most recent Cochrane review reported absence of evidence supporting potentially beneficial effects of combined interventions programs particularly for school children in primary education aged 6–12 years [40] over the BMI. Neither were statistically significant results observed in the same study when the studies were analyzed according to the duration and the combined intervention, whether it was less than one academic year or more than one academic year. Overall and similar to what we observed in our study combined diet and physical activity interventions are likely to produce either small to no change in BMI. The discrepancies observed between different reviews and meta-analyses may be due to variations in the selection criteria of the trials and, consequently, in the included ones, and to disparities in the outcomes considered.

Different studies have observed that although interventions in the school environment have effects on cardiac and cardiovascular health, they have no measurable effect on BMI [8,43]. Regarding the duration of the interventions, we did not observe differences in the subgroup analysis depending on whether the interventions lasted one year or longer. This result is in agreement with other previously published results that question [7] whether the duration of the school intervention has a determining effect on BMI.

When interpreting the results of studies that evaluate interventions on physical activity and dietary habits, one of the main problems we face is the lack of control over what children do beyond the school setting [44]. The effort made in the school environment may be adversely affected by the influence of the social environment, consumerism, and the media so that the effort undertaken in the school setting becomes diluted [45].

Our study stands out from similar studies previously published for including only randomized clinical trials, a precise age range, combined interventions of physical activity and dietary habits and, above all, the analysis of these interventions in the long-term in the school environment. These characteristics together reduce the likelihood of bias in the interpretation of the results reported.

It is noteworthy that most of the trials included in this review and meta-analysis had at least one source of bias among those analyzed, which generates uncertainty about the individual results and thus those of the meta-analysis. Furthermore, a significant percentage of cluster randomized clinical trials on interventions in the school setting for weight control do not address clustering effects, generally calculated by the intracluster correlation coefficient [46,47].

BMI was used as the only outcome measure in our study. This outcome measure was used from the point of view of feasibility since BMI is one of the main weight indices [41,48]. But both our study and others seem to indicate that the effectiveness of the proposed interventions to reverse the tide of the epidemic is small, primarily because so many social and environmental changes are driving the increase in childhood obesity and overweight, suggesting that overall the effect sizes associated with these interventions in the school setting fail to promote significant improvements in BMI [15].

We recognize different limitations in our study. Currently, only one of the included trials presented subgroup analyses according to sex. This diminishes our ability to assess whether there is a gender-dependent effect in the proposed interventions. There are a wide variety of countries included in the meta-analysis, so the effectiveness of BMI interventions may also be affected by the lifestyle and cultural patterns associated with each of these countries. Furthermore, the interventions proposed differ, and we acknowledge the potential for the existence of uncontrolled biases. The outcomes analyzed in this study have focused on BMI, which may lead to misleading results according to some authors, as it is rather unsusceptible to variations in children’s adiposity and is affected by age and sex [16,19,49]. Finally, differences in educational levels, although minimal between studies, may have affected the results.

Findings from our study indicate that long-term school-based interventions on physical activity and dietary habits received by children aged 6–12 years seem to have no effect on BMI. However, the promotion of such interventions should not be discouraged, as they promote additional positive health outcomes for other domains of children’s health. Future studies should take into account the possible gender-dependent effect of these interventions.

## Figures and Tables

**Table 1:** Pubmed search strategy.

Sequence	Terms/Combination
1	“obesity”[MeSH Terms]
2	“body weight changes”[MeSH Terms]
3	“obes *”[All Fields]
4	“weight gain”[All Fields] OR “weight loss”[All Fields]
5	“overweight”[MeSH Terms] OR “overweight”[All Fields] OR “overweighted” [All Fields] OR “overweightness”[All Fields] OR “overweights”[All Fields] OR “over weight”[All Fields] OR “overat*”[All Fields]
6	“bmi”[All Fields] OR “body mass index”[All Fields]
7	#1 OR #2 OR #3 OR #4 OR #5 OR #6
8	“diet therapy”[MeSH Terms]
9	“diet”[MeSH Terms] OR “diet”[All Fields] OR “diet”[MeSH Terms] OR
	“diet”[All Fields] OR “diets”[All Fields] OR “diet s”[All Fields] OR “dieted”[All Fields] OR
	“dieting”[All Fields] OR “diet”[MeSH Terms] OR “diet”[All Fields] OR “diets”[All Fields] OR
	“diet s”[All Fields] OR “dieted”[All Fields] OR “dieting”[All Fields]
10	“low calorie”[All Fields] OR “calorie control”[All Fields] OR “healthy eating”[All Fields]
11	“dietary fats”[MeSH Terms]
12	“fruit”[MeSH Terms] OR “fruit”[All Fields] OR “fruits”[All Fields] OR
	“fruit s”[All Fields] OR “fruited”[All Fields] OR “fruiting”[All Fields] OR “vegetable*”[All Fields]
13	“high fat*”[All Fields] OR “low fat *”[All Fields] OR “fatty food*”[All Fields]
14	#1 OR #8 OR #9 OR #10 OR #11 OR #12 OR #13
15	“exercise”[MeSH Terms]
16	“exercise therapy”[MeSH Terms]
17	“exercis*”[All Fields]
18	“aerobic”[All Fields] OR “aerobically”[All Fields] OR “exercise”[MeSH Terms] OR
	“exercise”[All Fields] OR “aerobics”[All Fields] OR “physical therapy”[All Fields] OR
	“physical activity”[All Fields] OR “physical inactivity”[All Fields]
19	“fitness class*”[All Fields] OR “fitness regime *”[All Fields] OR “fitness program*”[All Fields]
20	“physical training”[All Fields] OR “physical education”[All Fields]
21	#15 OR #16 OR #17 OR #18 OR #19 OR #20
22	“health promotion”[MeSH Terms]
23	“health education”[MeSH Terms]
24	“health promotion”[All Fields] OR “health education”[All Fields]
25	“school program *”[All Fields]
26	“school intervention *”[All Fields]
27	#22 OR #23 OR #24 OR #25 OR #26
28	#14 OR #21 OR #27
29	“child”[MeSH Terms]
30	“infant”[MeSH Terms]
31	“child *”[All Fields] OR “adolescen *”[All Fields] OR “infant *”[All Fields]
32	“teenage *”[All Fields] OR “young people”[All Fields] OR “young person”[All Fields]
33	“schoolchildren”[All Fields] OR “schoolchildren s”[All Fields] OR “school children”[All Fields]
34	“adolescent”[MeSH Terms]
35	#29 OR #30 OR #31 OR #32 OR #33 OR #34
38	#7 AND #35

**Table 2:** Description of the studies included in the systematic review and meta-analysis.

Reference	Study/Intervention Name	Country	Study Design	Age	Sex (Female)	Sample Size	Physical Activity Intervention	Diet Intervention	Duration of the Intervention
Grydeland 2014 [30]	HEIA	Norway	cluster-RCT	I: 11.2 (0.3) C:11.2 (03)	I: 50% C: 48%	I: 491 C:870	PA session once per week; provision of sports equipment to each class	A fruit and vegetable break once a week to eat fruit; posters for the classroom	20 months
Llargues 2012 [31]	AVall	Spain	cluster-RCT	I: 6.03 (0.3) C:6.03 (0.3)	I: 45.3% C:45.6%	I: 272 C:237	Every classroom used 3 h a week to develop activities related to health food habits and/or PA	Every classroom used 3 h a week to develop activities related to health food habits and/or PA	2 years
Magnusson 2012 [32]		Iceland	cluster-RCT	I: 7.3 (0.3) C: 7.4 (0.3)	I: 51% C: 60%	I: 128 C:138	PA intervention was progressive in nature, starting with approximately 30 min/day at the start of the study and increasing to approximately 60 min/day in the latter intervention year, where teachers who implement the intervention used various strategies to better integrate PA into the daily routine at school.	The main focus of the dietary intervention was on increasing fruit and vegetable intake, with both educational material and homework assignments. Food-based dietary guidelines on fish, fish liver oil and milk intake were also in focus, and parents, teachers, and school food service staffwere involved in the intervention.	2 years
Safdie 2013 [19]		Mexico	cluster-RCT	I: 9.7 (0.7) (plus) I: 9.7 (0.7) (basic) C: 9.8 (0.8)	I: 54% (plus) I:48.4% (basic) C: 48.6%	I: 224 (plus) I: 252 (basic) C:354	Basic: support to improve quality of PE lessons (in terms of amount of MVPA promoted). Mass communication and marketing were used to encourage children to be more physically active. In addition to the basic intervention, specialist PE/PE teachers who taught 1 extra PE class/week, and provided 15-min activity (calisthenics) sessions 4 times/week during morning recess. They also received additional financial investment to support the school’s efforts in implementing the intervention.	Basic: support to improve general environment (obesogenic environment) of the school, including the types of offering of foods and drinks (provided by external vendors) as snacks for the children at recess/break time. Additionally, mass communication and marketing was used to encourage children to eat healthy snacks, drink water instead of sugary drinks. Furthermore, schools received additional financial investment to support the effort to implement the intervention.	18-month
Xu 2017 [39]		China	cluster-RCT	I: 9.0 (1.4) C:9.0 (1.4)	I: 49.1% C:49.4%	I: 3476 C:3398	A classroom-based physical activity program for elementary students named “Happy 10” was used in PA intervention. The forms of PA includes games, dancing or rhythmic gymnastics, such as “invisible rope skipping”, “imitating animals”, and the “squat and multiplication table”, were linked with the core curriculum objectives and were conducted during breaks.	Courses on nutrition and health were given 6 times for the students, 2 times for the parents and 4 times for teachers and health workers. The school lunch cafeteria menu for students was evaluated periodically and specific nutrition suggestions were provided accordingly.	1 year
Kain 2014 [35]		Chile	cluster-RCT	I: 6.5 (1.1) C 6.7 (1.1) (Boys); I: 6.7 (1.1) C 6.5 (1) (Girls)	I: 44% C: 49%	I: 364 C: 423 (boys); I:287 C:400 (girls)	36 and 15 PE classes during the 1st semester and 56 and 32 classes during the 2nd semester	Classroom education consisted of a brief theoretical part and practical work in the form of activities like painting and puzzles	1 year
Kobel 2017 [3]	Join the Healthy Boat	Germany	cluster-RCT	I: 7.15 (0.66) C: 7.08 (0.66)	I: 53.1% C:48.8%	I: 318 C:207	The teachers are given read-to-use materials to provide one lesson per week (on physical activity, diet or screen media use) and daily exercise breaks of 10–15 min.	The main focus lies on the promotion of a healthy diet, especially targeting a reduction of soft drink consumption and an increase of fruit and vegetable intake	1 year
Liu 2019 [37]	ANGELO	China	cluster-RCT	I: 9.15 (0.75) C: 9.06 (0.58)	I:46.99% C:49.53%	I: 900 C:939	Children were encouraged to perform at least 60 min of moderate to vigorous physical activity (MVPA) each day.	Not to drink sugar-sweetened beverages or eat unhealthy snacks in schools, and drinking water was advocated. Improvement of school lunches.	1 year
Siegrist 2013 [38]	JuvenTUM	Germany	cluster-RCT	I + C 8.4 (0.7)	I + C 48%	I: 422 C:297	PE lessons: 45 min/month given by trained PE teachers (in addition to usual 2–3 45-min lessons given by usual teachers)	The dietary intervention focused on influencing the school setting by increasing the availability and healthy food choices with more vegetables and fruits and reducing the availability of foods that are energy-dense.	1 year
Siegrist 2018 [33]	JuvenTUM 3	Germany	cluster-RCT	I + C 11.1 (0.6)	I: 39.5% C:47%	I: 243 C:191	The intervention aimed to increase physical activity both inside and outside school by providing regular physical exercise in physical education classes and extra physical exercise at school essentially through active breaks during classes and active school recesses.	The intervention intended to encourage healthy eating patterns: fewer sugary beverages, healthier meals at school, and healthy breakfasts.	18 months
Waters 2018 [34]	Fun ‘n healthy in Moreland	Australia	cluster-RCT	I + C (5–12 at baseline)		I: 1320 C:1439	Increasing physical activity	Focus on increasing fruit, vegetable and water consumption,	3.5 years

I, intervention group; C, control group; PA, physical activity; PE, physical exercise; RCT, randomized controlled trial.

## Supplementary Materials

The following are available online at https://www.mdpi.com/article/10.3390/healthcare9040396/s1, Table S1: PRISMA 2009 Checklist, Table S2: Scopus and WOS search Syntax.
