# Supplementary material for: Long-Term Dietary and Physical Activity Interventions in the School Setting and Their Effects on BMI in Children Aged 6–12 Years: Meta-Analysis of Randomized Controlled Clinical Trials
Source: Healthcare (Basel). 2021 Apr 1;9(4):396. doi: 10.3390/healthcare9040396 (PMC8066711; doi:10.3390/healthcare9040396)
Supplement: Supplementary file 1 [file healthcare-09-00396-s001.zip › Suplementary Table 2.pdf]

Supplementary Materials

Scopus and WOS search Syntax

WOS <2009 to December 2019>; English

#	Search Statement	Results
**1**	TOPIC: (obesity)	**366183**
**2**	TOPIC: ("body weight changes")	**1329**
**3**	TOPIC: (obes*)	**443752**
**4**	TOPIC: ("weight gain" OR "weight loss")	**218333**
**5**	TOPIC: (overweight OR "over weight" or overat*)	**104108**
**6**	TOPIC: (bmi OR "body mass index")	**285449**
**7**	#6 OR #5 OR #4 OR #3 OR #2 OR #1	**757890**
**8**	TS=("diet therapy")	**1660**
**9**	TOPIC: (diet OR diets OR dieting)	**524161**
**10**	TOPIC: ("low calorie" OR "calorie control" OR "healthy eating")	**14.028**
**11**	TOPIC: ("dietary fats")	**2419**
**12**	TOPIC: (fruit or vegetable*)	**379458**
**13**	TOPIC: ("high fat*" OR "low fat*" OR "fatty food*")	**69798**
**14**	#13 OR #12 OR #11 OR #10 OR #9 OR #8 OR #1	**1179481**
**15**	TOPIC: (exercise)	**493006**
**16**	TOPIC: ("exercise therapy")	**4755**
**17**	TOPIC: (exercis*)	**493439**

18	TOPIC: (aerobics OR "physical therapy" OR "physical activity" OR "physical inactivity")	218427
19	TOPIC: ("fitness class*" OR "fitness regime*" OR "fitness program*")	1225
20	TOPIC: ("physical training" OR "physical education")	26335
21	#20 OR #19 OR #18 OR #17 OR #16 OR #15	655174
22	TOPIC: (health promotion)	62416
23	TOPIC: (health education)	233969
24	TOPIC: ("health promotion" OR "health education")	67080
25	TOPIC: ("school program*")	3490
26	TOPIC: ("school intervention*")	833
27	#26 OR #25 OR #24 OR #23 OR #22	285304
28	#27 OR #21 OR #14	1978346
29	TOPIC: (CHILD)	1753863
30	TOPIC: (INFANT)	425512
31	TOPIC: (CHILD* OR adolescen* OR infant*)	2485244
32	TOPIC: (teenage* OR "young people" OR "young person")	2536277
33	TOPIC: (schoolchildren OR "school children")	49804
34	TOPIC: (adolescent)	444281
35	#34 OR #33 OR #32 OR #31 OR #30 OR #29	2536277
36	#35 AND #7	125608
37	*TS=(randomised OR randomized OR randomisation OR randomisation OR placebo* OR (random* AND (allocat* OR assign*)) OR (blind* AND (single OR double OR treble OR triple))) NOT TS=(animal or animals or pisces or fish or fishes or catfish or catfishes or sheatfish or silurus or arius or heteropneustes or clarias or gariepinus or fathead minnow or fathead minnows or pimephales or promelas or cichlidae or trout or trouts or char or chars or salvelinus or salmo or oncorhynchus or guppy or guppies or millionfish or poecilia or goldfish or goldfishes or carassius or auratus or mullet or mullets or mugil or curema or shark or sharks or cod or cods or gadus or morhua or carp or carps or	1101142

	cyprinus or carpio or killifish or eel or eels or anguilla or zander or sander or lucioperca or stizostedion or turbot or turbots or psetta or flatfish or flatfishes or plaice or pleuronectes or platessa or tilapia or tilapias or oreochromis or sarotherodon or common sole or dover sole or solea or zebrafish or zebrafishes or danio or rerio or seabass or dicentrarchus or labrax or morone or lamprey or lampreys or petromyzon or pumpkinseed or pumpkinseeds or lepomis or gibbosus or herring or clupea or harengus or amphibia or amphibian or amphibians or anura or salientia or frog or frogs or rana or toad or toads or bufo or xenopus or laevis or bombina or epidalea or calamita or salamander or salamanders or newt or newts or triturus or reptilia or reptile or reptiles or bearded dragon or pogona or vitticeps or iguana or iguanas or lizard or lizards or anguis fragilis or turtle or turtles or snakes or snake or aves or bird or birds or quail or quails or coturnix or bobwhite or colinus or virginianus or poultry or poultries or fowl or fowls or chicken or chickens or gallus or zebra finch or taeniopygia or guttata or canary or canaries or serinus or canaria or parakeet or parakeets or grasskeet or parrot or parrots or psittacine or psittacines or shelduck or tadorna or goose or geese or branta or leucopsis or woodlark or lullula or flycatcher or ficedula or hypoleuca or dove or doves or geopelia or cuneata or duck or ducks or greylag or graylag or anser or harrier or circus pygargus or red knot or great knot or calidris or canutus or godwit or limosa or lapponica or meleagris or gallopavo or jackdaw or corvus or monedula or ruff or philomachus or pugnax or lapwing or peewit or plover or vanellus or swan or cygnus or columbianus or bewickii or gull or chroicocephalus or ridibundus or albifrons or great tit or parus or aythya or fuligula or streptopelia or risoria or spoonbill or platalea or leucorodia or blackbird or turdus or merula or blue tit or cyanistes or pigeon or pigeons or columba or pintail or anas or starling or sturnus or owl or athene noctua or pochard or ferina or cockatiel or nymphicus or hollandicus or skylark or alauda or tern or sterna or teal or crecca or oystercatcher or haematopus or ostralegus or shrew or shrews or sorex or araneus or crocidura or russula or european mole or talpa or chiroptera or bat or bats or eptesicus or serotinus or myotis or dasycneme or daubentonii or pipistrelle or pipistrellus or cat or cats or felis or catus or feline or dog or dogs or canis or canine or canines or otter or otters or lutra or badger or badgers or meles or fitchew or fitch or foumart or foulmart or ferrets or ferret or polecat or polecats or mustela or putorius or weasel or weasels or fox or foxes or vulpes or common seal or phoca or vitulina or grey seal or halichoerus or horse or horses or equus or equine or equidae or donkey or donkeys or mule or mules or pig or pigs or swine or swines or hog or hogs or boar or boars or porcine or piglet or piglets or sus or scrofa or llama or llamas or lama or glama or deer or deers or cervus or elaphus or cow or cows or bos taurus or bos indicus or bovine or bull or bulls or cattle or bison or bisons or sheep or sheeps or ovis aries or ovine or lamb or lambs or mouflon or mouflons or goat or goats or capra or caprine or chamois or rupicapra or leporidae or lagomorpha or lagomorph or rabbit or rabbits or oryctolagus or cuniculus or laprine or hares or lepus or rodentia or rodent or rodents or murinae or mouse or mice or mus or musculus or murine or woodmouse or apodemus or rat or rats or rattus or norvegicus or guinea pig or guinea pigs or cavia or porcellus or hamster or hamsters or mesocricetus or cricetulus or cricetus or gerbil or gerbils or jird or jirds or meriones or unguiculatus or jerboa or jerboas or jaculus or chinchilla or chinchillas or beaver or beavers or castor fiber or castor canadensis or sciuridae or squirrel or squirrels or sciurus or chipmunk or chipmunks or marmot or marmots or marmota or suslik or susliks or spermophilus or cynomys or cottonrat or cottonrats or sigmodon or vole or voles or microtus or myodes or glareolus or primate or primates or prosimian or prosimians or lemur or lemurs or lemuridae or loris or bush baby or bush babies or bushbaby or bushbabies or galago or galagos or anthropoidea or anthropoids or simian or simians or monkey or monkeys or marmoset or marmosets or callithrix or cebuella or tamarin or tamarins or saguinus or leontopithecus or squirrel monkey or squirrel monkeys or saimiri or night monkey or night monkeys or owl monkey or owl	

	monkeys or douroucoulis or aotus or spider monkey or spider monkeys or ateles or baboon or baboons or papio or rhesus monkey or macaque or macaca or mulatta or cynomolgus or fascicularis or green monkey or green monkeys or chlorocebus or vervet or vervets or pygerythrus or hominoidea or ape or apes or hylobatidae or gibbon or gibbons or siamang or siamangs or nomascus or symphalangus or hominidae or orangutan or orangutans or pongo or chimpanzee or chimpanzees or pan troglodytes or bonobo or bonobos or pan paniscus or gorilla or gorillas or troglodytes)	
**38**	#36 AND #37	**5673**

* Randomized Controlled Trials / Controlled Clinical Trials: Search Strategy adapted from the Cochrane ENT Group for Web of Science

Tjosvold, L. Randomized Controlled Trials / Controlled Clinical Trials: A Cut and Paste Search Strategy adapted from the Cochrane ENT Group for Web of Science. Adapted from “RCT Filters used by Cochrane ENT. Oxford (UK): Cochrane ENT Group; 2018.” John W. Scott Health Sciences Library, University of Alberta. Rev. January 14, 2021. Available from: https://docs.google.com/document/d/1aEVsIaeXVZY8_BND-sUB_19sTTMccgoxNUdznH54ilQ/edit.

Scopus <2009 to December 2019>; English; Controlled Study

#	Search Statement	Results
**1**	(obesity OR body AND weight AND changes OR obes* OR (weight AND gain OR weight AND loss) OR (overweight OR "over weight" OR overat*) OR bmi OR "body mass index") AND (diet AND therapy OR (diet OR diets OR dieting) OR "low calorie" OR "calorie control" OR "healthy eating" OR dietary AND fats OR fruit OR vegetable* OR "high fat*" OR "low fat*" OR "fatty food*") AND (exercise OR exercise AND therapy OR exercis* OR (aerobics OR "physical therapy" OR "physical activity" OR "physical inactivity") OR ("fitness class*" OR "fitness regime*" OR "fitness program*") OR ("physical training" OR "physical education")) AND (health AND	**2922**

	promotion OR health AND education OR ("health promotion" OR "health education") OR "school program*" OR "school intervention*") AND ((child OR infant) OR (child* OR adolescen* OR infant*) OR (teenage* OR "young people" OR "young person") OR (schoolchildren OR "school children") OR adolescent) View less	
